# ASHLEYS: automated quality control for single-cell Strand-seq data

**DOI:** 10.1093/bioinformatics/btab221

**Published:** 2021-04-01

**Authors:** Christina Gros, Ashley D Sanders, Jan O Korbel, Tobias Marschall, Peter Ebert

**Affiliations:** Center for Bioinformatics Saar, Saarland University, 66123 Saarbrücken, Germany; European Molecular Biology Laboratory (EMBL), Genome Biology Unit, 69117 Heidelberg, Germany; European Molecular Biology Laboratory (EMBL), Genome Biology Unit, 69117 Heidelberg, Germany; Institute for Medical Biometry and Bioinformatics, Heinrich Heine University, 40225 Düsseldorf, Germany; Institute for Medical Biometry and Bioinformatics, Heinrich Heine University, 40225 Düsseldorf, Germany

## Abstract

**Summary:**

Single-cell DNA template strand sequencing (Strand-seq) enables chromosome length haplotype phasing, construction of phased assemblies, mapping sister-chromatid exchange events and structural variant discovery. The initial quality control of potentially thousands of single-cell libraries is still done manually by domain experts. ASHLEYS automates this tedious task, delivers near-expert performance and labels even large datasets in seconds.

**Availability and implementation:**

github.com/friendsofstrandseq/ashleys-qc, MIT license.

**Supplementary information:**

Supplementary data are available at *Bioinformatics* online.

## 1 Introduction

Strand-seq is a single-cell short-read sequencing technique that assays sister chromatid inheritance patterns at the level of individual chromosomes (Falconer *et al.*, 2012; Sanders *et al.*, 2017). The Strand-seq protocol generates strand-specific sequencing libraries by labeling and later removing the non-template strand during DNA replication. The strand-of-origin information is recovered *in silico* as the read alignment either in Crick (C, forward) or in Watson (W, reverse) direction. The Strand-seq protocol affords unique insights in diverse applications, e.g. locating sister chromatid exchange events (Claussin *et al.*, 2017; Falconer and Lansdorp, 2013), characterizing complex genomic variation (Sanders *et al.*, 2016, 2020) or providing long-range phase information to assist genome assembly (Ghareghani *et al.*, 2018; Porubský *et al.*, 2017, 2020). Decreasing costs allow for studying larger cohorts, but the initial quality control (QC) step to discard low-quality libraries or control probes still requires human intervention. Domain experts manually evaluate and label each library in datasets comprising up to thousands of single cells. Replacing this laborious process with an automated tool for Strand-seq QC would be a prerequisite for further scaling of Strand-seq in the future. We developed a software for the *A*utomatic *S*election of *H*igh-quality *L*ibraries for the *E*xtensive anal*Y*sis of *S*trand-seq data (ASHLEYS). ASHLEYS is based on established machine learning technology and ships with ready-to-use classification models trained on a large cohort of Strand-seq libraries. ASHLEYS pretrained classifiers have been vetted on independent test data to ensure stable generalization performance on new Strand-seq data with similar feature characteristics. Next, we describe ASHLEYS’ feature model and summarize the performance of the default classifier recommended for QC of new Strand-seq libraries.

## 2 Materials and methods

**Fig. 1.**
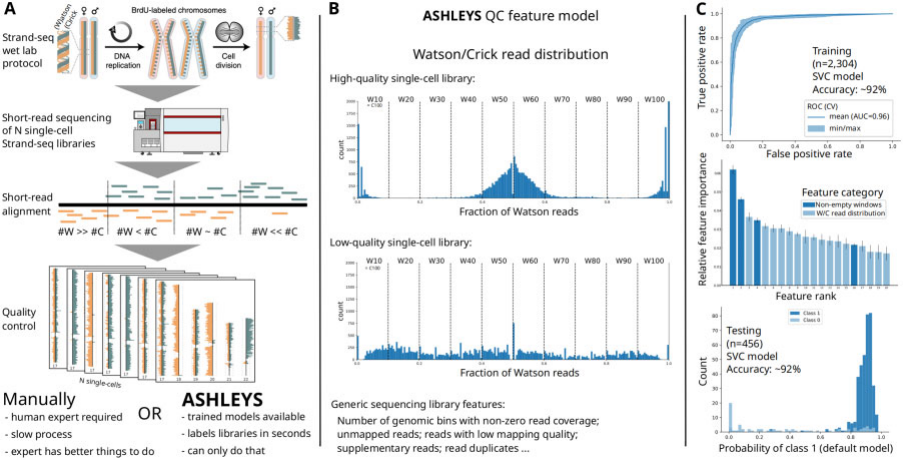
Summary of Strand-seq QC with ASHLEYS: (**A**) the Strand-seq protocol generates a large number of single-cell libraries that need to pass QC before downstream analysis. Strand-seq panels reused from Sanders *et al.* (2020; Fig. 1a). (**B**) Example for the Strand-seq specific feature of the Watson/Crick read distribution for high- (top) and low-quality (middle) libraries. ASHLEYS also evaluates library quality based on generic sequencing library features (bottom text). (**C**) Performance summary for the SVC model shipped with ASHLEYS for training and testing stages of model building. Feature names omitted in feature importance plot for improved readability (cf. Supplementary Fig. S1)

ASHLEYS’ main input is a set of BAM (Li *et al.*, 2009) files, one per single-cell paired-end Strand-seq library aligned to a reference genome. We provide a supporting pipeline (Supplementary Information) for data preprocessing following established examples (Fig. 1A) (Sanders *et al.*, 2017, 2020). ASHLEYS feature modeling uses statistics that describe either generic library QC characteristics, e.g. the number of unmapped reads or the number of low-quality alignments [default: MAPQ<10, Sanders *et al.* (2016, 2020)], or the W/C read distribution, which is a feature unique to Strand-seq data. Generic count features are normalized by the total library size to account for varying sequencing depth. The W/C feature computation is implemented as a sliding window approach covering a range of window sizes in a single run to capture technical artifacts at various size ranges (Supplementary Information). The window is shifted by half of its size in each step and ASHLEYS counts W and C reads per window. Due to the complementarity, only the W fraction of reads is stored. The feature is then modeled by binning the W fractions in steps of 0.1 and counting the number of windows per bin. The expectation for a high-quality library is to observe a W fraction of ≈0.5 for ≈50% of the windows, and closer to zero or one for ≈25% of the windows each, due to the random strand segregation during (diploid) cell division (Fig. 1B). Other common library issues lead to W/C signal ‘dropouts’ (Sanders *et al.*, 2017), which are modeled as the number of windows with non-zero W/C read coverage. The aggregated feature table for all libraries can then be used to train a new classifier, provided that expert labeling is available, or to predict quality labels using one of ASHLEYS pretrained models.

ASHLEYS is implemented as a Python3 tool using standard libraries for BAM processing (Pysam, github.com/pysam-developers/pysam) and machine learning (Pedregosa *et al.*, 2011), and includes a test dataset.

## 3 Models

ASHLEYS pretrained classifiers were tuned on a large dataset (*n* = 2304) generated as part of the Human Genome Structural Variation Consortium (HGSVC) (Chaisson *et al.*, 2019  Ebert, 2021 ). Model training including hyperparamter tuning and training error estimation was performed with 50 iterations of nested class-balanced 5-fold cross-validation (Fig. 1C, Supplementary Information). Model generalization performance was assessed on an independent test dataset (*n* = 456) labeled by the same domain expert (Sanders *et al.*, 2020). We recommend a linear support vector classifier (SVC) as default model for labeling new Strand-seq data. The SVC model shows consistently high performance on training (F1 score 93.9%, accuracy 91.6%) and on independent test data (F1 score 95.6%, accuracy 92.5%) (Fig. 1C), suggesting that the SVC is not overfitted to the training dataset.

In conclusion, ASHLEYS’ high performance and the resulting gains in efficiency facilitate scaling Strand-seq to even larger cohorts without burdening domain experts with an overwhelming amount of repetitive QC. This raises promising expectations for addressing further challenges such as extensive aneuploidy in cancer.

## Supplementary Material

btab221_Supplementary_Data
