# Supplementary material for: ASHLEYS: automated quality control for single-cell Strand-seq data
Source: Bioinformatics. 2021 Apr 1;37(19):3356–7. doi: 10.1093/bioinformatics/btab221 (PMC8504637; doi:10.1093/bioinformatics/btab221)
Supplement: btab221_Supplementary_Data [file btab221_supplementary_data.pdf]

Supplementary Information
ASHLEYS: automated quality control for single-cell Strand-seq data

Christina Eimer, Ashley D. Sanders, Jan O. Korbel, Tobias Marschall, Peter Ebert*

November 2020

*To whom correspondence should be addressed: peter.ebert@iscb.org

1 Supplemental Methods

1.1 Software

ASHLEYS is implemented as a Linux-only command line tool available at repository 1 (see below). The development environment was set up with `Python v3.7` (www.python.org), `Pysam v0.15.2` (github.com/pysam-developers/pysam) and `scikit-learn v0.23.2` (Pedregosa *et al.*, 2011). For an exact definition of the complete software environment, please refer to the environment file in the ASHLEYS repository under `environment/ashleys_env.yml`. The preprocessing pipeline that exemplifies short-read alignment, marking of duplicate reads and feature computation per Strand-seq library is available at repository 2 (see below). The preprocessing pipeline is implemented in the common workflow engine Snakemake (Köster and Rahmann, 2012), and we provide setup and usage instructions as part of the repository.

Repository URLs:
1. ASHLEYS: github.com/friendsofstrandseq/ashleys-qc
2. Preprocessing pipeline: github.com/friendsofstrandseq/ashleys-qc-pipeline

1.2 Feature modeling and model training

As introduced in the main text, ASHLEYS uses two feature categories as predictors of Strand-seq library quality: generic sequencing library features that are not Strand-seq specific and thus independent of the chosen window size(s) (see Table 1, rows 1–7 for detailed explanation), and a set of features that is derived from the binned Watson/Crick read distribution (see Table 1, rows 8–18). Hence, assuming ASHLEYS default window sizes $W = \{5, 2, 1, 0.8, 0.6, 0.4, 0.2\}$ (Mbp) are used, a fully trained model uses $7 + |W| * 11 = 7 + 7 * 11 = 84$ features to predict the quality of a Strand-seq library. The Strand-seq specific features are normalized using the total number of (non-empty) genomic windows; we emphasize here that the Watson/Crick ratio features (`W10` to `W100` in Table 1) are normalized using only the number of non-empty windows — and not the total number of genomic windows — because gaps in the reference or regions not amenable to short-read alignment will always result in a certain number of empty genomic windows (see Section 1.3 and Supplemental Figure 4). As a consequence, normalizing the Watson/Crick ratio features by the total number of genomic windows would distort the distribution shown in main Fig. 1B, whose expected shape for high-quality libraries is motivated by the strand segregation pattern during diploid cell division, and thus desirable to preserve in that form.

ASHLEYS default model that we recommend to label new Strand-seq libraries is a linear support vector classifier (SVC), and more specifically a trained instance of the `scikit-learn` class `sklearn.svm.SVC` (the trained model is available as a `pickle` dump in the ASHLEYS repository). The best hyperparameter setting for the SVC after 50 iterations of class-balanced nested cross-validation was determined to be $C = 10$ (training data split ratio in outer loop approximately 25%/75%, deviations result from enforcing class balance for the 75% split). Since a linear-kernel SVC allows for a straightforward interpretation of feature coefficients in terms of their importance in the classification process (in the following: feature importance), the relative feature importance was computed as the absolute feature coefficient normalized by the sum over all absolute feature coefficients. Supplemental Figure 1 lists the top 20 most importance features for the SVC default model (same as main Figure 1C where feature names were omitted for reasons of readability).

Feature	Explanation	Normalization	Depending on window size
unmap	reads that were not mapped to the reference genome	(unmap + map)	no
map	mapped reads	(unmap + map)	no
supp	supplementary reads, secondary reads, reads where quality control failed	(unmap + map)	no
dup	duplicate reads	(unmap + map)	no
mq	reads with mapping quality < 10	(unmap + map)	no
read2	paired-end reads of reverse direction	(unmap + map)	no
good	all resulting reads which are not counted in any of the previous categories	(unmap + map)	no
W10	windows with 0%-10% of Watson reads	total number of non-empty windows	yes
W20	windows with 10%-20% of Watson reads	total number of non-empty windows	yes
W30	windows with 20%-30% of Watson reads	total number of non-empty windows	yes
W40	windows with 30%-40% of Watson reads	total number of non-empty windows	yes
W50	windows with 40%-50% of Watson reads	total number of non-empty windows	yes
W60	windows with 50%-60% of Watson reads	total number of non-empty windows	yes
W70	windows with 60%-70% of Watson reads	total number of non-empty windows	yes
W80	windows with 70%-80% of Watson reads	total number of non-empty windows	yes
W90	windows with 80%-90% of Watson reads	total number of non-empty windows	yes
W100	windows with 90%-100% of Watson reads	total number of non-empty windows	yes
total	total number of non-empty windows	total number of windows	yes

Table 1: Summary of all predictive features used by ASHLEYS. The last column indicates whether the feature value depends on the chosen window size. By default, ASHLEYS uses window sizes $W = \{5, 2, 1, 0.8, 0.6, 0.4, 0.2\}$ Mbp.

1.3 Training and test data

ASHLEYS pretrained classifiers were tuned on a large data set (n=2,304) consisting of 1,146 high-, 140 medium- and 1,018 low-quality libraries generated by the HGSVC (see main text). Strand-seq libraries in the HGSVC data set consist of up to 47% "good" reads, i.e. reads that pass all preliminary quality checks and can be used to compute the Watson/Crick features (feature "`good`" in Table 1). On average, an HGSVC Strand-seq library contains 32% "good" reads. Model generalization performance was assessed on an independent test data set (n=456) consisting of 379 high- and 77 low-quality libraries labeled by the same domain expert (in the following: NBT data set) (Sanders *et al.*, 2020). The test libraries contain up to 45% "good" reads with an average of 19% "good" reads.

Supplemental Figures 2 and 3 complement main Figure 1B and exemplify visualizations for an HGSVC high-quality (Supplemental Figure 2) and a low-quality (Supplemental Figure 3) library that are currently used by human experts for manual quality evaluation of Strand-seq libraries. These example QC plots were produced with scripts that are part of the Mosaicatcher software (Sanders *et al.*, 2020). We also provide examples of distribution plots for some of the most important features of ASHLEYS' SVC default model (see Section 1.2 and Supplemental Figure 1) that illustrate differences between high- and low-quality Strand-seq libraries. Low-quality libraries show an overall tendency to a more dispersed feature distribution (Supplemental Figure 4), consistent with the Watson/Crick signal showing noise characteristics in low-quality libraries. For cases where low- and high-quality feature distributions seemingly overlap (e.g., feature `W100_0.2mb`), we commonly observe that only one class of libraries takes the lowest or highest possible value. In our example, Supplemental Figure 5 (rightmost panel) indicates that both low- and high-quality libraries cover a similar range of values, but only low-quality libraries (orange and light blue) actually take a value of 0.

2 Supplemental Figures

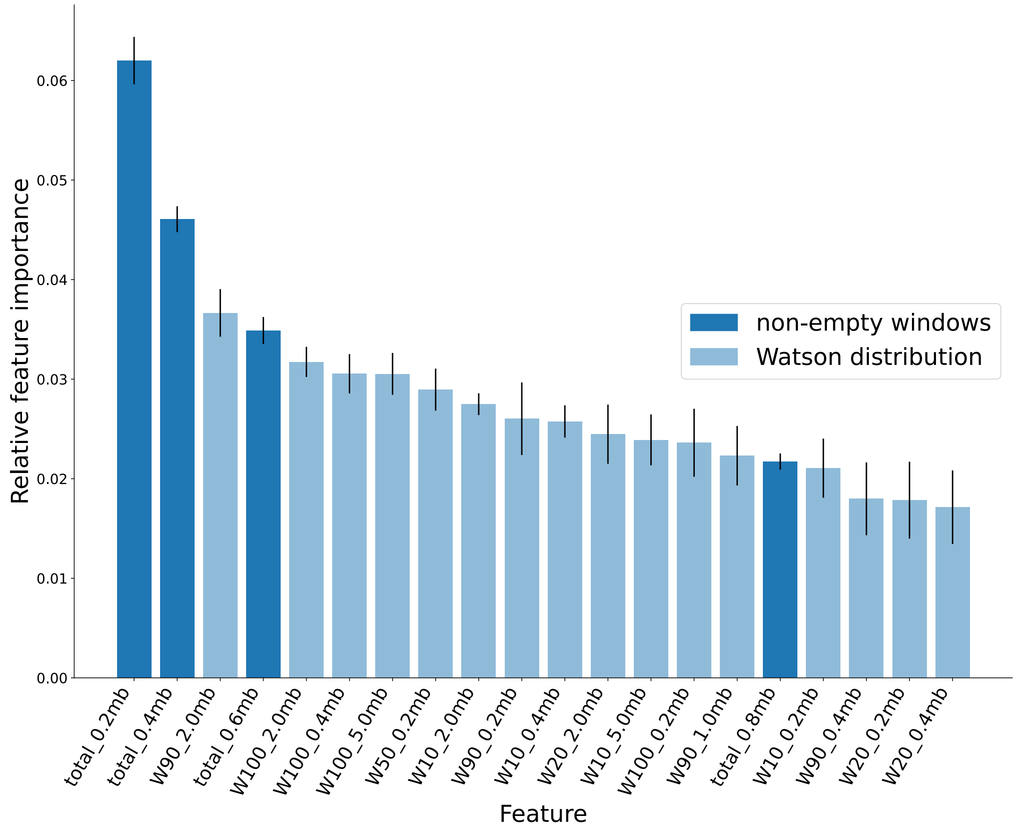

Figure 1: Feature importance of default SVC model with feature names. This plot is identical to main Figure 1C where feature names were omitted for reasons of readability.

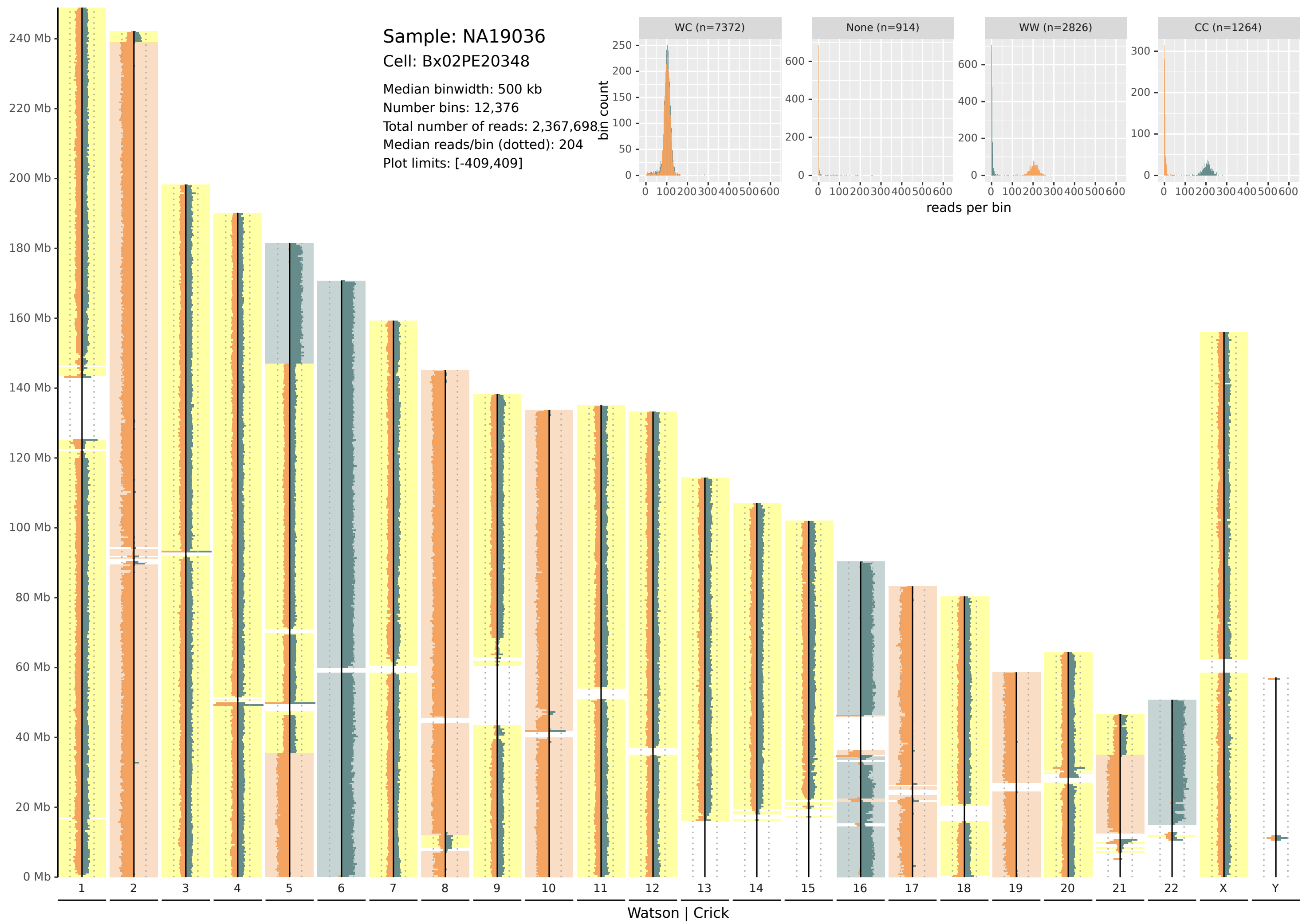

Figure 2: Example of an HGSVC high-quality library (2,367,698 reads): Watson/Crick read distribution is balanced in the majority of the genome (yellow background), and shows limited tendency to all Watson (orange background) or all Crick (teal background) reads; this observation conforms to the expectation of the strand segregation pattern during diploid cell division.

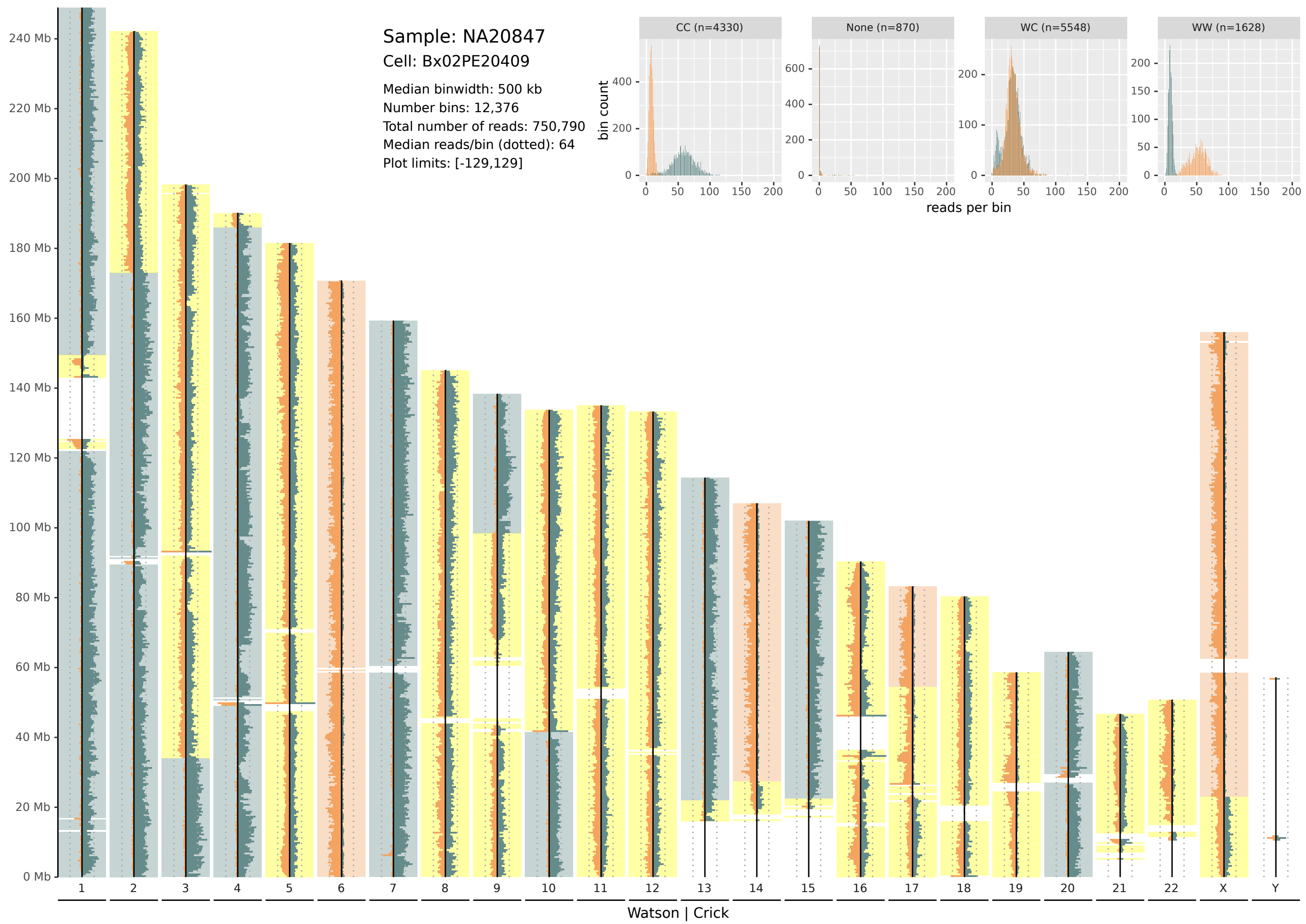

Figure 3: Example of an HGSVC low-quality library (750,790 reads): Watson/Crick read distribution is uneven and several regions exhibit signal of spurious alignments ("background noise"), e.g. on chromosome 7. A substantial fraction of the genome shows an unbalanced Watson/Crick read distribution (orange or teal background).

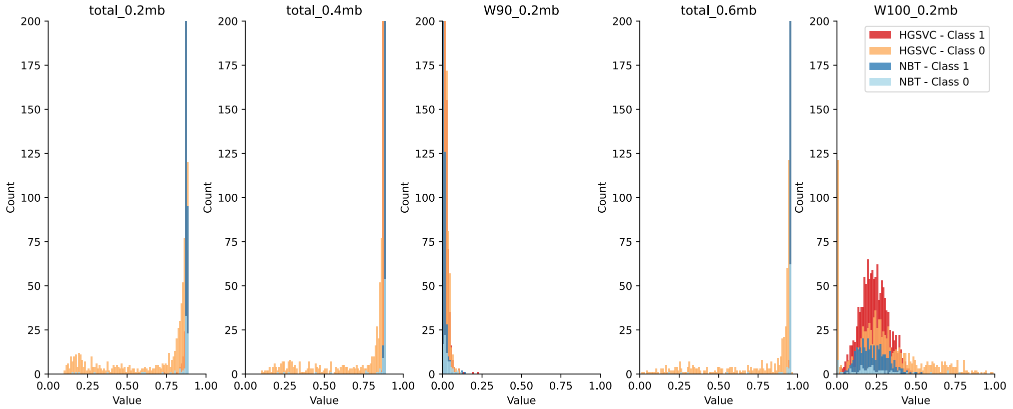

Figure 4: Feature distribution of HGSVC (training) and NBT (testing) data for the 5 most important features of the SVC model. Feature values (x-axis) for low- and high-quality class libraries (count, y-axis) are colored separately per data set. Capping below the value 1.0 for the features `total_0.2mb`, `total_0.4mb` and `total_0.6mb` is a result of necessarily empty genomic windows originating from, e.g., the chromosome centromere.

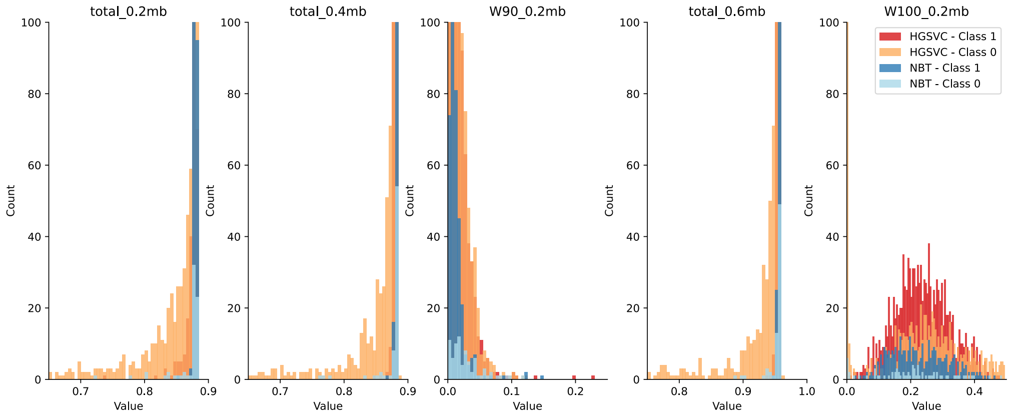

Figure 5: Detail of the x-axis of the previous feature distribution plot (Supplemental Figure 4). Feature values (x-axis) for low- and high-quality class libraries (count, y-axis) are colored separately for HGSVC and NBT data set.

References

Köster, J. and Rahmann, S. (2012). Snakemake–a scalable bioinformatics workflow engine. *Bioinformatics*, **28**(19), 2520–2522.

Pedregosa, F. *et al.* (2011). Scikit-learn: Machine learning in python. *Journal of Machine Learning Research*, **12**(85), 2825–2830.

Sanders, A. D. *et al.* (2020). Single-cell analysis of structural variations and complex rearrangements with tri-channel processing. *Nature Biotechnology*, **38**(3), 343–354.
